# Lingonberries alter the gut microbiota and prevent low-grade inflammation in high-fat diet fed mice

**DOI:** 10.3402/fnr.v60.29993

**Published:** 2016-04-27

**Authors:** Lovisa Heyman-Lindén, Dorota Kotowska, Elin Sand, Mikael Bjursell, Merichel Plaza, Charlotta Turner, Cecilia Holm, Frida Fåk, Karin Berger

**Affiliations:** 1 Department of Experimental Medical Science, Lund University, Lund, Sweden; 2 ImaGene-IT, Medicon Village, Lund, Sweden; 3 Astra-Zeneca R&D, Mölndal, Sweden; 4 Department of Chemistry, Center for Analysis and Synthesis, Lund University, Lund, Sweden; 5 Food for Health Science Centre, Lund University, Medicon Village, Lund, Sweden

**Keywords:** obesity, *Akkermansia*, hepatic steatosis, berries, metabolic endotoxemia, LBP, high-fat diet

## Abstract

**Background:**

The gut microbiota plays an important role in the development of obesity and obesity-associated impairments such as low-grade inflammation. Lingonberries have been shown to prevent diet-induced obesity and low-grade inflammation. However, it is not known whether the effect of lingonberry supplementation is related to modifications of the gut microbiota. The aim of the present study was to describe whether consumption of different batches of lingonberries alters the composition of the gut microbiota, which could be relevant for the protective effect against high fat (HF)-induced metabolic alterations.

**Methods:**

Three groups of C57BL/6J mice were fed HF diet with or without a supplement of 20% lingonberries from two different batches (Lingon1 and Lingon2) during 11 weeks. The composition and functionality of the cecal microbiota were assessed by 16S rRNA sequencing and PICRUSt. In addition, parameters related to obesity, insulin sensitivity, hepatic steatosis, inflammation and gut barrier function were examined.

**Results:**

HF-induced obesity was only prevented by the Lingon1 diet, whereas both batches of lingonberries reduced plasma levels of markers of inflammation and endotoxemia (SAA and LBP) as well as modified the composition and functionality of the gut microbiota, compared to the HF control group. The relative abundance of *Akkermansia* and *Faecalibacterium*, genera associated with healthy gut mucosa and anti-inflammation, was found to increase in response to lingonberry intake.

**Conclusions:**

Our results show that supplementation with lingonberries to an HF diet prevents low-grade inflammation and is associated with significant changes of the microbiota composition. Notably, the anti-inflammatory properties of lingonberries seem to be independent of effects on body weight gain.

The increasing prevalence of obesity is a worldwide health problem closely linked to diet and lifestyle factors. Obesity and its metabolic complications, such as non-alcoholic fatty liver disease (NAFLD), insulin resistance and dyslipidemia, contribute to a higher risk of developing type 2 diabetes. Accumulating evidence suggests that low-grade chronic inflammation is a common denominator for these metabolic diseases (1), and recent research demonstrates the important role of the gastrointestinal tract in contributing to this subclinical inflammation (2). The gut and the composition of the gut microbiota are crucial for nutrient handling and energy harvest and influence whole-body metabolism, immune response and insulin sensitivity (3–7). The metabolic endotoxemia and associated inflammation observed in obesity are proposed consequences of a dysfunctional gut barrier resulting in leakage of lipopolysaccharide (LPS) and proinflammatory cytokines into the circulation (5, 7). The importance of the interaction between the diet and the gut is demonstrated by studies in mice showing that high-fat (HF) diet–induced inflammatory changes in the intestine develop before the onset of obesity and other metabolic complications (6).

Lingonberries (*Vaccinium vitis-idaea* L.) are commonly consumed in Scandinavia and have attracted increasing interest due to their nutritional properties (8) and putative role as a food with beneficial health effects (9–13). Recently, it was found that lingonberry supplementation prevents weight gain and associated negative effects of HF diet consumption in mice (9); however, several questions regarding the cause of the preventive effect remain to be further investigated. High antioxidant and antimicrobial activities of lingonberries have been described (14–17) and might be of particular relevance for interactions with the gastrointestinal milieu. The present study was conducted to investigate if supplementation with different batches of lingonberries modifies the gut microbiota of HF diet fed C57BL/6J mice, as this may be an important factor to assess in order to promote understanding of the metabolic effects of lingonberry intake.

## Research design and methods

### Preparation and analysis of diets

HF diet (control) and HF diets supplemented with 20% (w/w) of freeze-dried lingonberries were prepared by Research Diets (New Brunswick, NJ, USA). The HF diet and HF diet supplemented with lingonberries from Batch1 (referred to as Lingon1) have been characterized and described previously (9), and the HF diet has been shown to induce obesity, insulin resistance and low-grade inflammation compared to a low-fat diet (9, 10). In this study, an independent second batch of lingonberries (Batch2) was obtained from the same provider that supplied Batch1 (MOLDA AG, Dahlenburgh, Germany). Batch2 was used to manufacture a second lingonberry diet, referred to as Lingon2. All diets contained 45% of kcal from fat, 20% kcal from protein and 35% kcal from carbohydrate and were designed to have matching nutrient content (Table 1). After manufacturing, all diets were analyzed for fiber content (Table 1), and Lingon1 and Lingon2 were subjected to detailed analyses of fatty acids, cholesterol, benzoic and sorbic acid (Eurofins, Lidköping, Sweden). Phenolic compounds in the lingonberry diets were extracted using a previously optimized method of pressurized hot water extraction (18). Total antioxidant capacity was measured using two different methods non-specific to phenolic compounds (the Folin-Ciocalteu assay (19) and the trolox equivalent antioxidant capacity (TEAC) assay (20)) to take into account potential different antioxidative mechanisms present in the lingonberry diets. The content of different subclasses of phenolic compounds was analyzed by setting up a reverse phase high-performance liquid chromatography (RP-HPLC) method coupled to diode array (DAD), electrochemical (ECD) and charge aerosol (CAD) detectors as previously described (21), with minor modifications. In brief, for performing analysis of anthocyanins in the present study, the mobile phase consisted of (A) 60 mM ammonium formate buffer (pH 1.5) in water, and (B) methanol with a higher concentration of formic acid (5%) compared to the previous study. Based on the information provided by DAD, the analysis of the separated compounds using selected wavelengths of 280, 350 and 520 nm allowed identification of phenolic compounds, flavonols and anthocyanins, respectively. The CAD detector was used to carry out semi-quantification of the phenolic compounds found in the lingonberry diets, as CAD is a universal detector and the response of these compounds does not depend on the chemical structure. In addition, ECD was used to estimate the antioxidant capacity of the phenolic compounds present in the lingonberry diets.

**Table 1:** Composition of the experimental diets^a^

	Control	Lingon1	Lingon2
Calculated energy (kcal)			
Protein	812.0	812.0	812.0
Carbohydrate	1422.4	1422.4	1422.4
Starch	731.2	731.2	723.2
Sucrose	347.2	347.2	347.2
Fructose	172.0	172.0	172.0
Glucose	172.0	172.0	180.0
Fat	1822.5	1822.5	1822.5
Total kcals	4,057	4,057	4,057
Calculated energy per gram diet (kcal/g)	4.5	4.3	4.4
Calculated energy (kcal%)			
Protein	20	20	20
Carbohydrate	35	35	35
Fat	45	45	45
Analyzed fiber (g/100 g diet)^b^			
Insoluble fiber	10.1	7.9	8.7
Soluble fiber	<1	1.6	1.2
Total fiber	10.5	9.5	9.9

a The diets are formulated to have matched macronutrient composition by energy (Research Diets, NB, USA).

b Fiber analyzed by Eurofins, Lidköping, Sweden.

### Animals and study design

The study was approved by the Animal Ethics Committee in Lund, Sweden (Permit Number: M185-11) in accordance with the Council of Europe Convention (ETS 123). Male C57BL/6JBomTac mice, 6 weeks old with an average body weight of 23.8±1.0 g, were obtained from Taconic (Skensved, Denmark). The animals were housed in a controlled environment (12 h light–dark cycle, 7 am–7 pm). After 9 days of acclimatization the mice were separated into three groups based on mean body weight per cage and housed in groups of 5 mice per cage. Mice were fed Lingon1 diet, Lingon2 diet or HF diet (*n=*10 mice/diet group) for 11 weeks *ad libitum*. At week 7, 8 and 10 weeks post diet introduction, the mice were placed in clean cages on grids and feces was collected over 24 h. At the end of the study, 4 h fasted animals were anesthetized with an intraperitoneal injection of midazolam (Midazolam (5 mg/mL), Panpharma S.A., Luitré, France) and a mixture of fluanisone 10 mg/mL and fentanyl citrate 0.315 mg/mL (Hypnorm, VetaPharma, Leeds, UK). Body composition was determined by dual-energy X-ray absorptiometry (DEXA) using a Lunar PIXImus (GE Lunar, Madison, WI, USA). Blood samples were taken by intraorbital puncture and animals were sacrificed by cervical dislocation and selected tissues were dissected, weighed and saved for further analysis.

### Body weight, food intake and fecal energy

Body weight and food intake were monitored weekly. The energy intake was expressed per mouse to adjust for the loss of one mouse in Lingon2 during week 9 of the study. Estimated mean intake per mouse was calculated as follows: [weekly food consumption (kcals) per group (mean of 2 cages/group)]/[number of mice per diet group]. The energy content of dried feces collected at week 7, 8 and 10 was determined using a bomb calorimeter (C6000, IKAWerke GmbH, Germany) and expressed as energy content of excreted feces during 24 h per cage. Ingested digestible energy intake was established by calculating the mean daily energy intake at week 7, 8 and 10 related to the energy excreted in feces sampled at the same time points. Feed efficiency was obtained by calculating body weight gain per calories consumed and not excreted into feces.

### Plasma analysis and assessment of insulin resistance

Plasma levels of triacylglycerol, total cholesterol, high density lipoprotein (HDL)-cholesterol, alanine aminotransferase (ALT), glucose, insulin and homeostasis model assessment-estimated insulin resistance (HOMA-IR) were determined as previously described (9). Serum amyloid A (SAA) and LPS-binding protein (LBP) were measured in plasma using commercial ELISA-kits (Tridelta Development Ltd, Wicklow, Ireland and Nordic Biosite, Täby, Sweden).

### Real-time PCR of intestine tissue

Jejunum (*n*=6–7) was snap-frozen and ground to a powder in a mortar under liquid nitrogen. The powder was subjected to total RNA extraction and reverse transcription followed by quantitative PCR (qPCR) analysis as previously described (22). Expression of *Tlr4* (toll-like receptor 4, forward: GCCTTTCAGGGAATT; reverse: AGATCAACCGATGGA), *Occludin* (forward: ATGTCCGGCCGATGC; reverse: TTTGGCTGCTCTTGG) (DNA Technologies A/S, Aarhus, Denmark), *Emr1* (EGF-like module-containing mucin-like hormone receptor-like, Mm00802529_m1), *Gcg/proglucagon* (Mm01269055_m1), *Reg3g* (regenerating islet-derived protein 3 gamma, Mm00441127_m1) and *Muc2* (mucin 2, Mm01276696_m1) (Applied Biosystem, Foster City, CA, USA) were quantified and related to the expression of the reference gene *Actb* (beta-actin, Mm00607939_s1).

### Immunocyto- and histochemistry in liver and adipose tissue

The medial lobe of the liver and epididymal visceral fat (*n*=3 per group) were dissected, fixated in 4% paraformaldehyde, embedded in paraffin (SVA, Uppsala, Sweden) and sectioned (5 µm) for immunocyto- and histochemistry to illustrate effects on hepatic steatosis and inflammatory cell infiltration in adipose tissue. To study liver steatosis and Kupffer cells in the liver, paraffin sections were deparaffinized, hydrated and rinsed in hydroxymethylaminomethane (TRIS) buffer (pH 7.6). To eliminate endogenous peroxidase activity and unspecific background staining, sections were exposed to 0.3% hydrogen peroxide containing TRIS buffer for 20 min and Protein K (code: S3004, Dako, Glostrup, Denmark) for 6 min before being treated with 2% bovine serum albumin for 20 min. Sections were incubated with an antibody against the surface-specific glycoprotein F4/80 for macrophages (dilution 1:50, ST-MCA497R, Nordic Biosite, Täby, Sweden) over night at 4°C, before being treated with biotinylated anti-rat IgG (code 4001; dilution 1:50; Vector BA) for 30 min. The VECTASTAIN ABC kit (code PK-6100; Vector Laboratories Inc., CA, USA) (DAB; code ab 644238; Abcam, Cambridge, MA, USA) was used in combination with hydrogen peroxide in accordance with manufacturer's instructions to visualize the biotinylated F4/80. The sections were counter stained with Mayer's hematoxylin (Histolab, Göteborg, Sweden) before being dehydrated, mounted and scanned using a computerized image analyzing system Imagescope (Aperio Scan Scope, Vista, CA, USA). The F4/80 immunoreactive macrophages stained brown. Microvesicular steatosis was evaluated according to Brunt et al. (23), with a few minor modifications. Hepatocyte nuclei, macrophages and macrovesicles were analyzed with ImageJ 1.49 (Canadian content interactive media, Ontario, Canada). The analysis procedure was automated using a Macro program specifically developed for the project. Five images each from zone1 and 3 were collected per animal. Zone1, located around the portal triads, and zone3, located around central veins, were analyzed separately due to their functional differences. Hepatocyte nuclei, macrophages and macrovesicles were identified and quantified, giving the objects per mm^2^ in each image. To study inflammatory infiltration in visceral fat, paraffin sections were stained with hematoxylin and eosin (H&E), scanned and analyzed in Imagescope (Aperio Scanscope) and the number of leukocytes per mm^2^ were quantified using Image J (24).

### Sequencing and analysis of bacterial 16S rRNA genes

Whole cecum was dissected, weighed and snap-frozen in liquid nitrogen (*n=*9–10). DNA extraction, PCR amplification and sequencing were performed at GATC Biotech AG, Konstanz, Germany. Briefly, the cecal tissue and content were thawed on ice and DNA was extracted using the QIAamp DNA Stool Mini Kit (Qiagen, Hilden, Germany), with a bead beating step included. The V1-3 region of 16S rRNA genes were amplified by PCR with forward and reverse primers containing Illumina adapter sequences and unique dual indexes used to tag each PCR product (25): 27F (5′-AGAGTTTGATCCTGGCTCAG-3′) and 534R (5′-ATTACCGCGGCTGCTGG-3′). Paired-end sequencing with a read length of 2×250 bp was carried out on a Miseq instrument using a Miseq reagent kit v2 (Illumina, San Diego, CA, USA). Sequences were analyzed with the free software package Quantitative Insights into Microbial Ecology (QIIME) using default parameters, except where specified (26). Sequences were removed if they were shorter than 200 nucleotides or longer than 1,000 nucleotides and contained ambiguous bases, primer mismatches, homopolymer runs in excess of six bases or uncorrectable barcodes. Similar sequences were binned into operational taxonomic units (OTUs) using UCLUST (27), with a minimum pairwise identity of 97%. The most abundant sequence in each OTU was chosen to represent its OTU. Representative sequences from each OTU were aligned using PyNAST (a python-based implementation of NAST in QIIME (28)) and taxonomy was assigned using the Greengenes (29) database (v. 13_8) and the RDP classifier (30) using a minimum percent identity of 90%.

### Statistical analysis of bacterial 16S rRNA genes

GraphPad Prism 6 software (GraphPad Software, San Diego, CA, USA) was used to identify significant differences in bacterial relative abundances between groups using one-way ANOVA and Tukey's test to adjust for multiple comparisons at each taxonomic level. Further, alpha- and beta-diversity were analyzed in QIIME, using a non-parametric *t*-test and Bonferroni correction for multiple comparisons and the ANOSIM and Adonis non-parametric statistical tests, respectively. To investigate whether bacterial taxa could be identified as biomarkers related to lingonberry intake, Linear Discriminant Analysis (LDA) of effect size (LEfSe) was applied on the OTU table according to Segata et al. (31) The online software tool PICRUSt was applied on the OTU table to infer the functional capacity from 16S rRNA gene sequencing data, and resulting significant pathways were collapsed into three levels of pathways and visualized using the LEfSe cladogram with a LDA score>3 (32).

### Statistical analysis

Unless stated otherwise, data are displayed as mean±SD and analyzed by one-way ANOVA followed by Tukey's multiple comparisons test. In cases where Gaussian distribution could not be assumed, groups were compared using Kruskal–Wallis post test. The ROUT test (33) was performed to statistically identify outliers with 99% confidence level. Statistical analyses were performed using GraphPad Prism 6.0 and differences with a *p*-value<0.05 were considered significant.

## Results

### Lingonberry supplementation affects body weight, metabolic and inflammatory plasma parameters

**Fig. 1:**
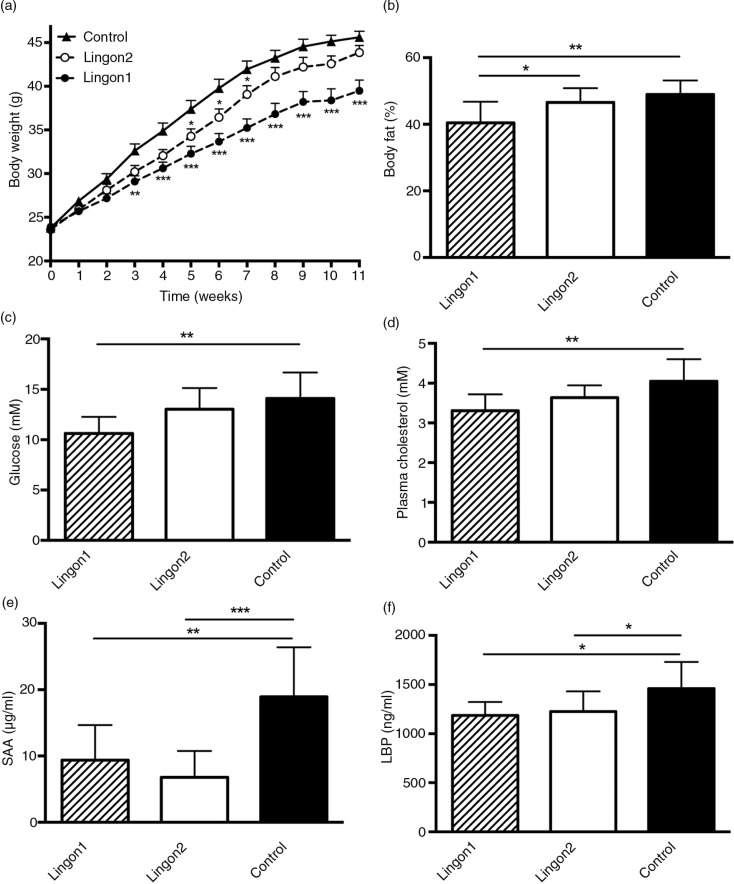
Metabolic and inflammatory characteristics of mice fed high-fat diet (control) supplemented with different batches of lingonberries. (a) Lingon1 (black circles) and Lingon2 (white circles) supplementation had effects on body weight gain compared to the control (black triangles). The stars next to Lingon1 and Lingon2 data points denote significant differences compared to the control group for each week (two-way ANOVA mean±SEM; Dunnett's post-hoc test). The percentage of body fat (b), plasma levels of glucose (c) and cholesterol (d) were reduced by supplementation with Lingon1. (e) The plasma concentration of the inflammatory marker serum amyloid A (SAA) and (f) the endotoxemia marker LPS-binding protein (LBP) was reduced by both lingonberry diets compared to the control. Values represent mean±SD, *n=*9–10. One-way ANOVA; Tukey's post-hoc test *p<0.05, **p<0.01 or ***p<0.001.

After 11 weeks, the mice receiving HF diet supplemented with Lingon1 weighed 39±3.9 g, which was significantly lower (*p*=0.0003) compared to the control group receiving HF diet without berries (46±2.2 g) (Fig. 1a). Mice in the Lingon2 group weighed less than mice in the control group at week 5, 6 and 7, but the final weight of 44±2.8 g was not significantly different from the control group (*p=*0.43). In addition, there was a significant difference in body weight between Lingon1 and Lingon2 (*p=*0.01). The percentage of body fat was significantly lower in the Lingon1 group compared to Lingon2 and control (Fig. 1b). There were no significant differences in the lean body mass or weight of epididymal fat pads amongst the groups (data not shown). Cecum weight was higher (*p<*0.0001) in the Lingon1 (0.65±0.09 g) and Lingon2 (0.62±0.13 g) groups compared to the control (0.23±0.03 g). In the Lingon1 group, the fasting plasma levels of glucose and cholesterol were significantly lower compared to the control group (Fig. 1c and d), whereas there was only a tendency (*p=*0.056) towards reduced glucose levels in the Lingon2 group. The HDL-cholesterol levels in the Lingon1 group (1.8±0.23 mM) were significantly lower (*p=*0.018) compared to control (2.1±0.28 mM), whereas the change in HDL-cholesterol in the Lingon2 group was not statistically significant (1.9±0.19 mM, *p*>0.05). There were no significant differences in plasma insulin levels (Lingon1: 1,059±848.7 pM; Lingon2: 1,547±1,622 pM; Control: 1,933±1,730 pM) (*n*=9–10). The calculated HOMA-IR index was not significantly different between the groups (data not shown), however it tended to be lower in Lingon1 compared to the control. SAA and LBP in plasma reflect inflammation and LPS-levels, respectively, and were significantly reduced in plasma from mice receiving Lingon1 and Lingon2 compared to the control group (Fig. 1e and f).

### Diet composition and food intake

The analysis of the general composition of the Lingon1 and Lingon2 diets did not reveal any difference in the content of compounds such as total fiber, insoluble fiber, specific fatty acids, cholesterol, benzoic and sorbic acid (data not shown). According to the margin of error of the utilized methods, the only indicated difference was in soluble fiber content (Table 1). Data illustrating energy intake, fecal energy content, ingested digestible energy and feed efficiency of mice receiving the different diets are presented in Table 2. In order to measure the antioxidant capacity, quantify the phenolic composition and analyze phenolics in the lingonberry diets by subclass, a HPLC–DAD–ECD–CAD method was set up, and the obtained data are summarized in Table 3. The Lingon1 and Lingon2 diets presented similar total antioxidative capacity with both *in vitro* assays; however, the antioxidative capacity was slightly higher in Lingon1 than in Lingon2 according to the Folin–Ciocalteu assay (Table 3).

**Table 2:** Energy intake and fecal energy content

	Lingon1	Lingon2	Control
Energy intake^a^ (kcal/mouse)	84.1±3.8	89.6±4.5	94.0±5.0
Fecal energy content^b^ (kcal/24 h/cage)	11.7±0.9	10.6±1.1	8.2±0.5
Ingested digestible energy^c^ (kcal/day)	80.2±1.7	82.68±0.7	87.2±1.1
Feed efficiency of digestible energy^d^ (g/kcal*10^6^/day)	0.18±0.15	0.25±0.19	0.22±0.08

a Mean weekly energy intake expressed per mouse.

b Mean fecal energy content excreted per cage per 24 h sampled at week 7, 8 and 10.

c Mean of ingested energy – fecal energy.

d Mean weekly body weight gain per consumed energy per cage.

Values are represented as group mean±SD.

**Table 3:** Phenolic composition of lingonberry diets

	Lingon1	Lingon2
Total phenolic compounds (total peak area/g sample)^a^	0.61±0.02	0.73±0.06
Antioxidant contribution of phenolics (total peak area/g sample)^b^	1.08±0.05	1.10±0.04
Phenolic composition (total peak area/g sample)^c^		
Phenolic compounds (280 nm)	9.08±0.67	9.60±0.11
Flavonols (350 nm)	1.62±0.11	1.78±0.13
Anthocyanins (520 nm)	3.15±0.16	3.40±0.19
Total antioxidant capacity		
Folin-Ciocalteu assay (mg GAE/g sample)	0.967±0.115	0.764±0.081
TEAC assay (mmol trolox/ g sample)	0.025±0.001	0.024±0.002

a Calculated by charge aerosol detection (CAD).

b Calculated by electrochemical detection (ECD).

c Calculated by diode array detection (DAD).

Values are represented as mean±SD.

### Histology analysis of lingonberry-mediated effects on liver and adipose tissue inflammation

**Fig. 2:**
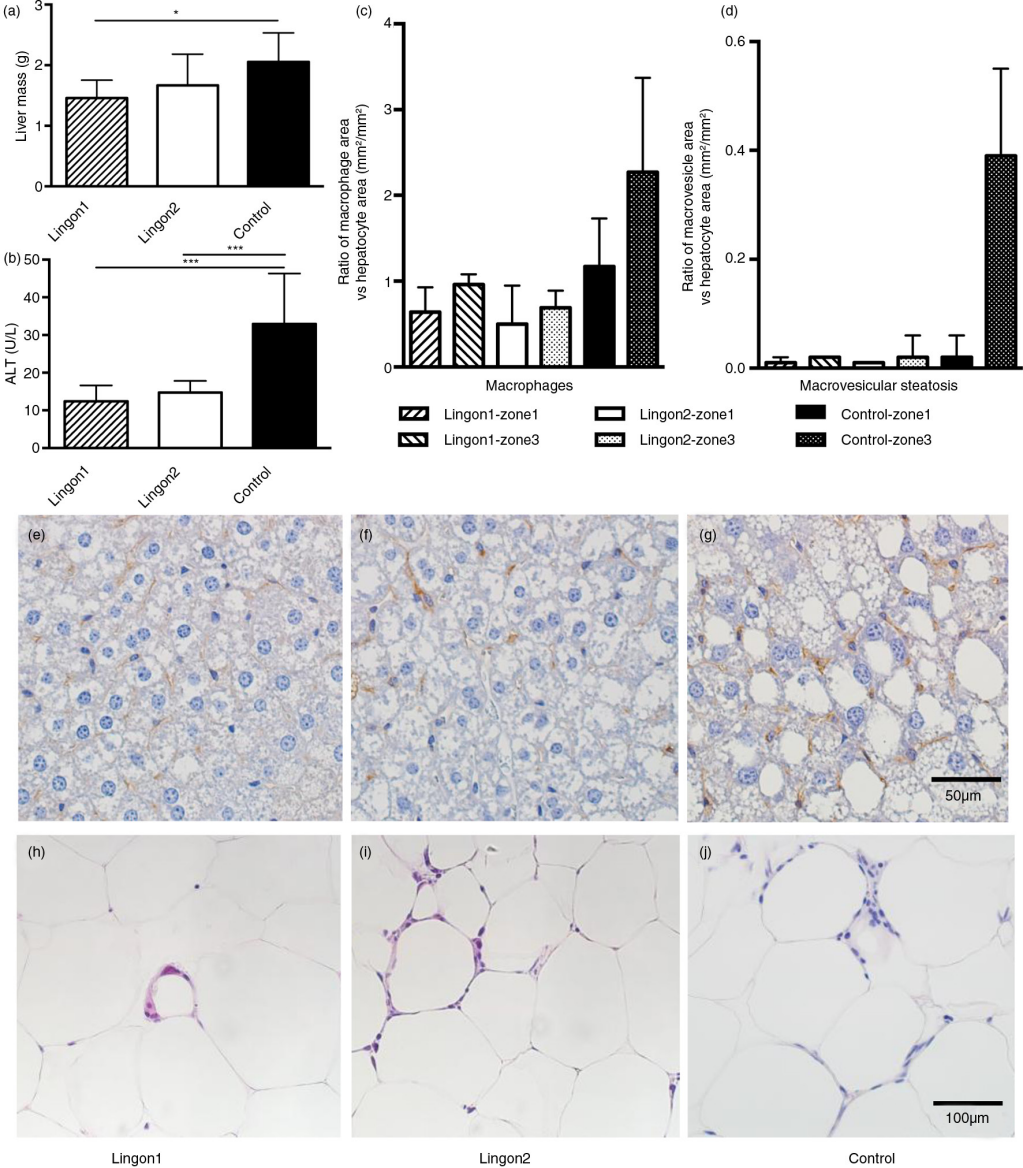
The effect of lingonberry supplementation on liver steatosis and inflammation. (a) Liver mass was significantly lower in mice supplemented with lingonberries from batch1 (Lingon1) compared to mice receiving HF without berries (Control). (b) The plasma levels of ALT, a marker for liver dysfunction, were reduced in groups receiving both Lingon1 and Lingon2 compared to control (mean±SD, *n=*8–10), *p<0.05 or ***p<0.001. The livers and epididymal fat pads from three mice per group were subjected to histological analysis. Quantification of slides revealed differences in the relative prevalence of macrophages (c) and macrovesicular steatosis (d) between groups, as well as between different liver zones within the groups; data plotted as median and interquartile range (*n=*3, two sections analyzed per group, five areas per zone). Representative slides of livers stained with an antibody specific for macrophages (in brown) and counterstained with hematoxylin are shown in (e) Lingon1, (f) Lingon2 and (g) Control. (h)–(j) displays hematoxylin and eosin stained epididymal adipose tissue with leukocyte nuclei forming crown-like structures around adipocytes.

The average mass of the livers in group Lingon1 was significantly reduced compared to the control (Fig. 2a). The group receiving Lingon2 diet displayed a tendency (*p=*0.12) to reduced liver mass compared to the control group. The plasma levels of ALT, a marker of liver dysfunction, were significantly reduced by both lingonberry diets compared to control mice not receiving lingonberries (Fig. 2b). The histochemical analysis of liver is illustrated by one representative slide per group in Fig. 2e–g. The evaluation according to Brunt et al. (23) graded the microvesicular steatosis as absent or mild in Lingon1, mild or marked in Lingon2 and marked in all samples in the control group. The data generated using ImageJ to analyze area of hepatocytes, macrophages and macrovesicular steatosis are visualized in Fig. 2c and d as the area occupied by macrophages (brown stained) and macrovesicles related to the area occupied by hepatocytes. The relative area occupied by macrophages appeared to be higher in livers from the control group, especially in the zone3 area around the central vein, compared to mice receiving lingonberries. The area of macrovesicular steatosis was relatively similar amongst the groups in zone1, surrounding the portal triads. In zone3, the macrovesicular steatosis was 20-fold higher in the control group compared to Lingon1 and Lingon2. In epididymal adipose tissue, histochemistry revealed a higher number of leukocytes, most of them arranged in crown-like structures, in the control group (median: 177±22 counts/mm^2^) compared to mice receiving Lingon1 (121±26 counts/mm^2^) and Lingon2 (138±47 counts/mm^2^) (representative slides displayed in Fig. 2h–j).

### Lingonberry supplementation affects expression of genes involved in intestinal barrier function and inflammation

**Fig. 3:**
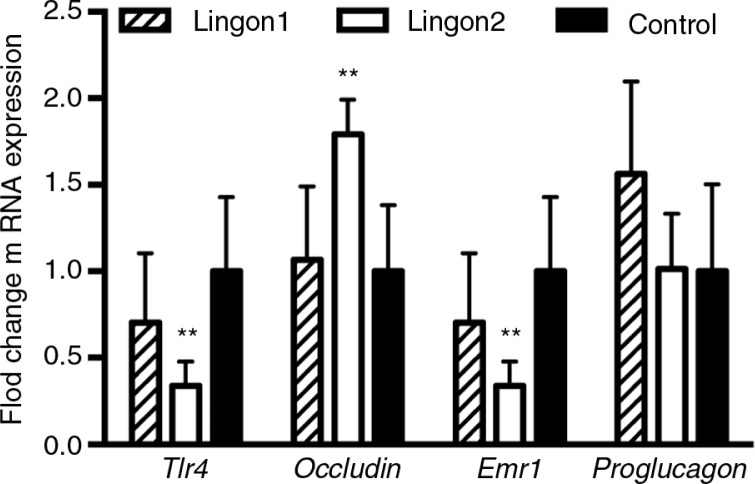
Intestinal gene expression of markers related to inflammation and gut barrier integrity. The fold change of jejunal mRNA levels of indicated genes are displayed relative to the expression in the control group. Lingon2 significantly increased expression of the gene for Tlr4 (LPS-stimulated receptor), the tight-junction protein occludin and the macrophage marker Emr1 (F4/80). Values significantly different from control are depicted **p<0.01 (one-way ANOVA), mean±SD, *n=*6–7.

The results from the jejunal gene expression analysis are displayed in Fig. 3. Compared to the control group, Lingon2 group displayed decreased expression of LPS-sensing *Tlr4* and macrophage marker *Emr1*, and increased expression of the tight-junction protein-encoding *occludin* gene. There was a tendency to increased expression of *Gcg* (proglucagon) (*p=*0.07) in the Lingon1 group compared to control, whereas there were no significant differences between the groups in expression of *Reg3g* and *Muc2* (data not shown).

### Effects of lingonberry intake on the composition and functionality of cecal gut microbiota

**Fig. 4:**
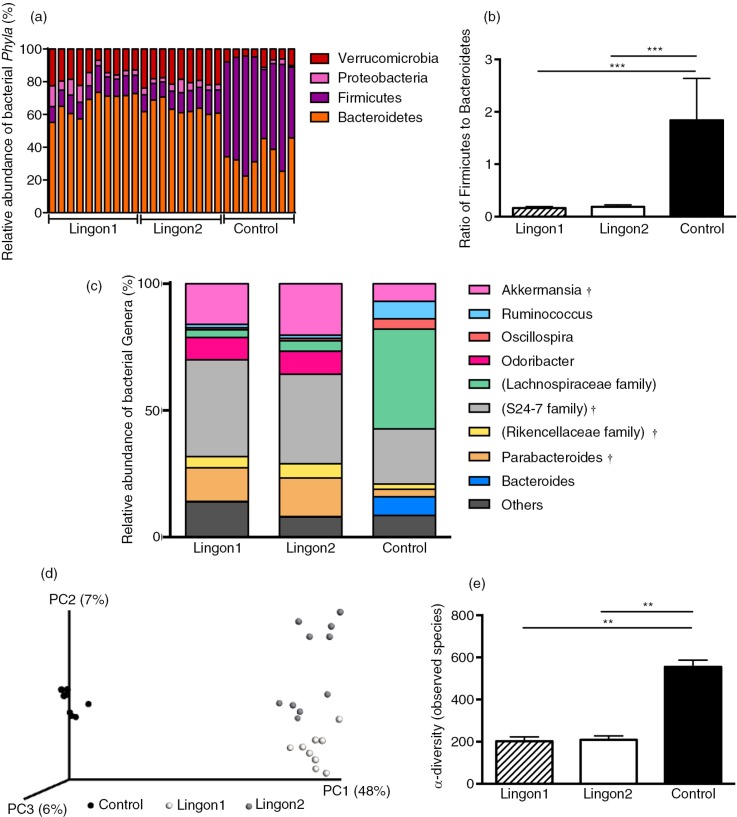
Lingonberry intake promotes modification of gut microbiota composition in high-fat fed mice. Cecal gut microbiota was analyzed using 16S rRNA sequencing in mice fed high-fat diets for 11 weeks. Two experimental groups were fed high-fat diet supplemented with two different batches of lingonberries (Lingon1 and Lingon2) and were compared to a group receiving high-fat diet without berries (Control). (a) Bars represent the relative abundance (%) of bacterial phyla and the Firmicutes/Bacteroidetes ratio is displayed in (b). (c) The relative abundance of bacterial genera (%) that were significantly modified by lingonberry supplementation (*p*<0.0001) and had a relative abundance above 4% in one or more of the groups. † denotes genera significantly different between the Lingon1 and Lingon2 groups (*p*<0.05) and each color represents a separate genera. In cases where the genus was unclassified, the family name is written in parenthesis. (d) Unweighted PCA plot showing the degree of bacterial taxonomic similarity between samples at the genus level; the larger the distance between samples, the more different they are with respect to the axes (PC1, PC2 and PC3). Lingon1: white circles; Lingon2: grey circles; Control: black circles. (f) The alpha-diversity was decreased in groups receiving lingonberries compared to the control group (non-parametric *t*-test and Bonferroni correction). *n=*8–10, mean±SD, **p<0.01, ***p<0.0001.

**Fig. 5:**
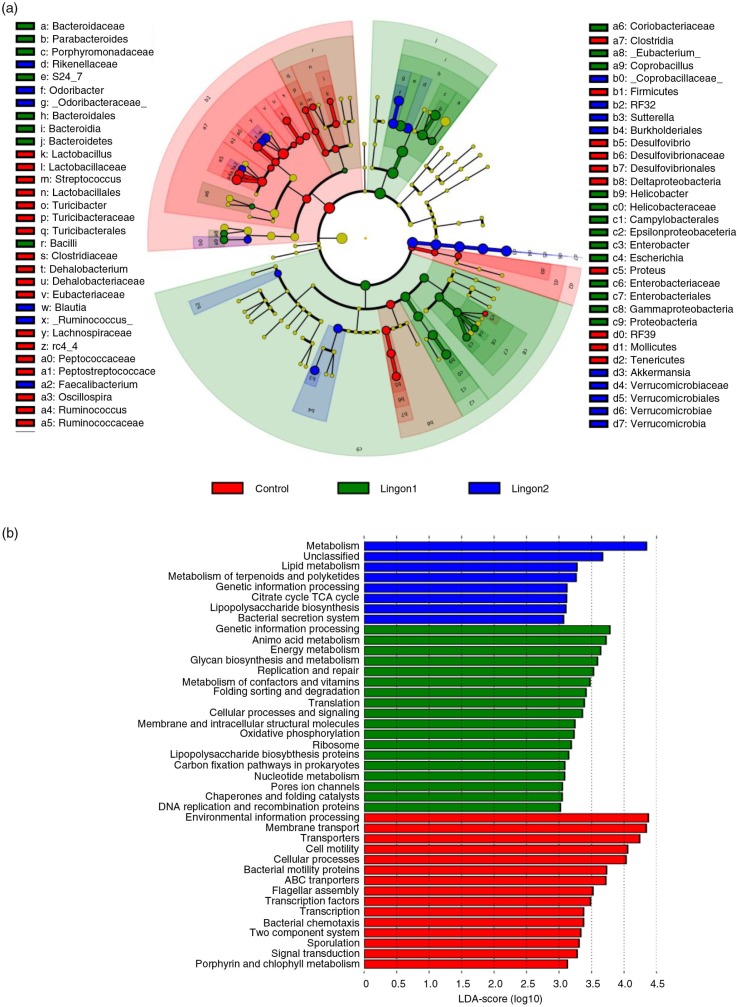
Taxonomic and functional diversity of the cecal microbiota of mice receiving high-fat diet with or without supplementation with lingonberries. (a) Significant changes in relative abundance (proportional to circle size) are marked in red (elevated in control), green (elevated in Lingon1) and blue (elevated in Lingon2). Data are derived using LEfSe and differences with a LDA score greater than 2 are considered significant, whereas non-significant changes are marked with yellow. Going towards the center of the figure, letter symbols represent phylum, class, order, family and genus, which are identified in the legend. (b) Metabolic pathways (KEGG) altered by diet were identified using PICRUSt and LEfSe (LDA>3.0). Pathways enriched in the microbiota of mice in the control group are displayed in red, Lingon1 in green and Lingon2 in blue.

After quality filtering, a total number of 9,125,002 sequences were generated, with an average of 314,655 sequences per sample in the dataset. Two samples in the control group failed to produce sequences and were excluded from further analyses. Remaining samples had between 229,369 and 377,540 sequences per sample. The overall composition of the bacterial community was influenced by diet (Fig. 4d), with significant alterations between lingonberry diets and the control group in the phyla Bacteroidetes, Firmicutes, Verrucomicrobia and Proteobacteria (Fig. 4a). The abundance of Proteobacteria was only significantly different between the control and the Lingon1 group (*p*<0.01), and Verrucomicrobia differed between lingonberry groups and control (*p*<0.0001), as well as between the Lingon1 and Lingon2 group (*p<*0.01). Foremost, analysis at the phylum level showed that the relative abundance of Bacteroidetes was significantly increased and the relative abundance of Firmicutes was decreased by lingonberry supplementation (*p<*0.0001) (Fig. 4a). The Firmicutes/Bacteroidetes ratio was significantly reduced by both Lingon1 and Lingon2 diet compared to the control (*p<*0.0001) (Fig. 4b). At genus level, 14 bacterial taxa differed significantly between the control and lingonberry groups, and five taxa differed between Lingon1 and Lingon2 (*p*<0.05, significantly different genera with a relative abundance >4% in at least one group are displayed in Fig. 4c. The HF diet–induced increase in Firmicutes was largely accounted for by increase of reads assigned to an unclassified genus within the *Lachnospiraceae* family and to a smaller extent from the genera *Ruminococcus* and *Oscillospira* (Fig. 4c). The increase of Bacteroidetes in the lingonberry groups was to a large extent caused by increased relative abundance of bacteria belonging to an unclassified genus in the *S24-7* family (Fig. 4c). The genus *Parabacteriodes* was also significantly increased in the Lingon1 (relative abundance 15%) and Lingon2 (13%) groups compared to the control group (3%) (Fig. 4c). The genus *Odoribacter* was not present in the control group, but had a relative abundance of approximately 9% in the lingonberry groups (Fig. 4c). Furthermore, the genus *Akkermansia*, belonging to the Verrucomicrobia phylum, was significantly increased in both lingonberry groups compared to the control (*p<*0.0001, relative abundance 7%) and *Akkermansia* was also significantly higher in the Lingon2 group (20%) compared to the Lingon1 group (16%) (*p<*0.0001) (Fig. 4c). The alpha-diversity, analyzed with the observed species test, was higher in the control group (554±32) compared to Lingon1 (203±22) and Lingon2 (209±19) (*p=*0.003) (Fig. 4e). Unweighted UniFrac analysis (Fig. 4d) revealed that supplementation with lingonberries promoted modification of gut microbiota and PC1 explained 48% of the observed variation (*p<*0.001, ANOSIM). In addition, the second principal component explained 7% of the variation in microbiota community, which accounts for the difference in microbiota driven by supplementation with lingonberries from different batches (Lingon1 vs. Lingon2). LEfSe analysis confirmed the described changes in the cecal microbiota of mice in the Lingon1 and Lingon2 groups and further identified additional bacterial genera related to the control, Lingon1 or Lingon2 groups of mice (Fig. 5a). The comparison of functional pathways enriched in the control, Lingon1 and Lingon2 groups (Fig. 5b) revealed an enrichment of genes belonging to pathways related to metabolism in the Lingon-groups, and an enrichment of genes involved in transport and motility in the control group.

## Discussion

There is growing evidence that increased consumption of fruit and vegetables reduces the risk of chronic disease such as cardiovascular disease and stroke, and may also prevent body weight gain and development of type 2 diabetes (34). The protection of fruits and vegetables against obesity-related disorders has partly been attributed to polyphenols, and intake of flavonoid-rich foods, such as berries (35), has been associated with a lower risk of developing type 2 diabetes (36, 37). In agreement with a previous study (9), we show that supplementation with lingonberries (Lingon1) prevents HF diet–induced weight gain, increased liver weight, body fat accumulation and elevated plasma levels of glucose and cholesterol. Surprisingly, we found that supplementation with a different batch of lingonberries (Lingon2) did not have the same capacity to attenuate weight gain. Nevertheless, both batches of lingonberries altered the gut microbiota composition and were effective in preventing HF-induced low-grade inflammation and endotoxemia, demonstrating that the effects of lingonberries on these parameters are independent of effects on body weight.

The gut microbiota may partly influence host metabolism by leakage of proinflammatory factors into the circulation, such as LPS, which may trigger inflammatory response in the liver and other tissues (38, 39). In our study, intake of both batches of lingonberries led to reduced plasma levels of LBP, which indicates reduced endotoxemia compared to mice receiving HF diet (40). SAA proteins are acute phase proteins secreted by the liver in response to inflammatory stimuli, including LPS (41), and mice receiving lingonberry diets displayed a marked reduction in the plasma levels of SAA compared to control. Furthermore, HF feeding as well as increased LPS and SAA-levels are associated with lipid accumulation into the liver, which may contribute to development of liver inflammation and hepatic insulin resistance. Even though only mice in the Lingon1 group had reduced liver size compared to the control, both lingonberry batches prevented a rise in plasma ALT and tended to reduce hepatic macrovesicular steatosis and the presence of macrophages. These results suggest that liver function was improved by lingonberry supplementation to an HF diet, and are in line with previous studies showing that lingonberry supplementation reduces HF-induced hepatic lipid accumulation (9). Interestingly, the findings presented here demonstrate that both batches of lingonberries protected against HF-induced liver steatosis and inflammation, which may be important for preventing development of systemic low-grade inflammation. Notably, as only one batch of lingonberries prevented diet-induced obesity, we show that the anti-inflammatory effect of lingonberries is more than a result of reduced body weight. Furthermore, as germ-free C57BL/6J mice are protected against HF-induced liver triglyceride accumulation and have reduced plasma levels of SAA (42) one might speculate that interaction with the gut is an involved mechanism in the metabolic effects of lingonberries.

Our study shows that supplementation of HF diet with lingonberries leads to profound changes in the cecal microbiota structure, including a decrease in the ratio of Firmicutes to Bacteroidetes. A high Firmicutes/Bacteroidetes ratio is characteristic for obesity-driven dysbiosis and associated with HF diet consumption, and the reduction of Firmicutes/Bacteroidetes ratio observed in response to lingonberries is similar to what have been reported previously in lean mice and upon dietary modifications including intake of low-fat diets and polyphenol supplementation (43–45). In the present study, HF diet induced an increase of unclassified members of the *Lachnospiraceae* family as well as the genera *Ruminococcus* and *Oscillospira*. Increased abundance of the same groups of bacteria has been found to associate with promotion of diabetes pathogenesis in the NOD mouse model of type 1 diabetes (46). In the same model, an increase in unclassified bacteria belonging to the *S24-7* family correlated to changes in gut immune parameters and was associated with protection against development of diabetes. Furthermore, bacteria assigned to the family *Lachnospiraceae* and genus *Bacteroides* have been shown to decrease in response to dietary treatments preventing HF-induced metabolic syndrome (44, 47), and species belonging to *Lachnospiraceae* have been linked to development of obesity and type 2 diabetes in *ob/ob* mice (48). Consequently, the fact that lingonberries promoted an increase in *S24-7*, as well as a decrease in *Lachnospiraceae*, *Ruminococcus, Oscillospira* and *Bacteroides*, indicates that lingonberries may confer beneficial effects on the gut microbiota composition in relation to diabetes development.

Analysis of bacteria at the genus level revealed that the relative abundance of the genus *Akkermansia* was increased in response to lingonberry consumption. The genus *Akkermansia* mainly consists of the mucin-associated species *Akkermansia muciniphila*, which has been associated with healthy gut mucosa (49, 50) and has attracted considerable interest as a potentially beneficial gut bacterium. For example, administration of *A. muciniphila* reverses HF diet–induced endotoxemia, adipose tissue inflammation and insulin resistance in C57BL/6 mice (51), potentially by improving the mucus layer function and thereby preventing toxin translocation. Moreover, the presence of *A. muciniphila* inversely correlates with body weight in rodents and humans (22, 52, 53). In the present study, we found that supplementation of HF diet with the Lingon2 batch increased the levels of *Akkermansia* more (+4%) than the Lingon1 batch, and LEfSe analysis ranked the increase of *Akkermansia* as the strongest biomarker of Lingon2 supplementation. This finding is notable as only the Lingon1 batch reduced body weight gain. Generally, our results are similar to studies in the same mouse model where supplementation with polyphenols (44) or cranberry extracts (47) prevented negative metabolic effects of HF diet and increased abundance of *A. muciniphila*. However, in the present study the increase of *Akkermansia* in response to lingonberries was independent of body weight. Furthermore, the genus *Faecalibacterium* was identified as a marker for Lingon2 supplementation, which is of interest as the butyrate-producing species *F. Prausnitzii* has anti-inflammatory properties in both mice and humans (54, 55). However, these observations require further studies to determine the importance of specific bacteria for the effects of lingonberries.

We also addressed the impact of lingonberries on the functionality and metabolic activities of the gut microbiota. We found that the microbiome in the HF control group had an increased abundance of genes belonging to categories such as transporters, ABC transporters, membrane transporters, cell motility, bacterial motility proteins, bacterial chemotaxis, flagellar assembly, two component system, transcription and signal transduction compared to the lingonberry groups. Notably, the same gene categories were found by Hildebrandt et al. to be increased in the fecal microbiome of mice fed HF diet compared to mice fed standard chow (56). The observed enrichment of genes for transporters is paralleled by results from Turnbaugh et al. (45) where a Western diet induced an enrichment of genes for transporters and ABC transporters in the cecal microbiota of humanized gnotobiotic mice compared to mice receiving a low-fat diet. Furthermore, in our study, the microbiome in mice receiving lingonberries was enriched with genes related to metabolism of energy, lipids, amino acids, and nucleotides. Similar findings were reported by Hildebrandt et al. in mice fed chow diet. The authors hypothesized that an HF-induced increase of nutrient transporters and a decrease of metabolic genes may be an adaptation to lower amounts of nutrients reaching the colonic microbiota, thus favoring bacteria with increased numbers of nutrient transporters (56). Although more research is needed to interpret the implications of these shifts in functionality, it appears clear that supplementing HF diet with lingonberries has a large impact on both gut microbiota composition and functionality.

Gut permeability is controlled by tight-junction proteins such as occludin, which is a proposed key marker of tight-junction integrity (38). In the present study, *occludin* was significantly upregulated in jejunum in response to the Lingon2 diet compared to the HF control. This is in line with studies describing decreased expression of *occludin* in response to HF feeding in mice (57), whereas treatment with polyphenols (44) and cranberry proanthocyanidines (58) restores *occludin* expression and gut barrier function, respectively. The effect on *occludin* could be of importance for the action of the Lingon2 diet, as barrier integrity and gut leakage seem to be linked to the negative metabolic effects of HF feeding (7, 57) and obesity-associated low-grade inflammation (59). Moreover, the genes encoding toll-like receptor 4 (*Tlr4*) and the macrophage marker F4/80 (*Emr1*) were downregulated in mice receiving Lingon2 diet compared to the control. TLR4 activation in response to fatty acids or LPS is implicated as yet another mechanism by which HF diet and dysbiosis induce impaired barrier function, low-grade inflammation and endotoxemia with detrimental effects on whole-body metabolism (57). Kim et al. described that HF diet increased expression of intestinal *Tlr4* and inflammatory mediators, whereas tight-junction proteins were decreased, compared to lean C56BL/6J mice fed a low-fat diet (57). However, as no effects on intestinal markers were observed in response to Lingon1, it seems that this mechanism is not a major contributor to the capacity of lingonberries to prevent HF-induced low-grade inflammation observed by both lingonberry diets, at least not in the jejunum.

The ability of lingonberries in batch Lingon1 to prevent weight gain has been described before (9) and was replicated in this study, whereas the new lingonberries in batch Lingon2 did not significantly prevent HF-induced weight gain and adiposity. In contrast to the previous study, we here observe a tendency to reduced energy intake in mice supplemented with lingonberries, especially Lingon1. There is also a tendency to increased energy content in feces from mice receiving lingonberries, potentially caused by fiber or due to less digestion and absorption of nutrients. Studies in humans suggest that lingonberries may delay the digestion of sucrose (60), and there is a need for additional studies describing how lingonberries affect absorption and digestibility, since these processes may be influenced by gut microbiota and phytochemical intake (61).

Our data show that the antioxidative capacity as well as phenolic and nutrient composition of the two lingonberry diets is similar. Nonetheless, the reason for the difference in metabolic outcomes in response the and Lingon2 diets is intriguing, and should be the subject for further in-depth investigations. The quantity of nutrients and secondary metabolites in plants is highly variable and depends on several factors including cultivar, growth conditions, time of harvest and environmental exposures (62, 63). It has been theorized that new plant tissues produce defense compounds that affect growth and metabolism of for example microorganisms (e.g. acids and flavonoids), whereas more ripe tissues produce digestibility reducers that act on general digestive processes (64, 65); however, extensive further research is required to identify which factors and compounds define specific metabolic effects of lingonberries. The presented results highlight that variability between different batches is an important factor to take into consideration in nutritional research.

## Conclusions

To the best of our knowledge, this is the first work describing that lingonberry intake modifies the gut microbiota composition and prevents endotoxemia and low-grade inflammation. The present study replicates the previous finding that lingonberries exert anti-obesity effects when supplemented to an HF diet; however, the magnitude of the effect varies depending on the batch of berries. Regardless of the berry batch and effect on body weight, lingonberries promote modifications of the gut microbiota and protects against low-grade inflammation, including reduced inflammation in liver and adipose tissue. Specifically, lingonberries decrease the Firmicutes/Bacteroidetes ratio and the relative abundance of *Akkermansia* independently of changes in body weight. We propose that modification of the gut microbiota is important for the anti-inflammatory effect of lingonberries, and that lingonberries should be further investigated for its potential role in dietary strategies to prevent metabolic disease.
